# Estimates of early outbreak-specific SARS-CoV-2 epidemiological parameters from genomic data

**DOI:** 10.1073/pnas.2308125121

**Published:** 2024-01-04

**Authors:** Timothy G. Vaughan, Jérémie Scire, Sarah A. Nadeau, Tanja Stadler

**Affiliations:** ^a^Department of Biosystems Science and Engineering, Eidgenössiche Technische Hochschule Zurich, Basel 4058, Switzerland; ^b^Computational Evolution Group, Swiss Institute of Bioinformatics, Lausanne 1015, Switzerland

**Keywords:** epidemiology, phylodynamics, birth–death

## Abstract

We estimate the basic reproductive number and case counts for 15 distinct Severe acute respiratory syndrome coronavirus 2 (SARS-CoV-2) outbreaks, distributed across 11 populations (10 countries and one cruise ship), based solely on phylodynamic analyses of genomic data. Our results indicate that, prior to significant public health interventions, the reproductive numbers for 10 (out of 15) of these outbreaks are similar, with median posterior estimates ranging between 1.4 and 2.8. These estimates provide a view which is complementary to that provided by those based on traditional line listing data. The genomic-based view is arguably less susceptible to biases resulting from differences in testing protocols, testing intensity, and import of cases into the community of interest. In the analyses reported here, the genomic data primarily provide information regarding which samples belong to a particular outbreak. We observe that once these outbreaks are identified, the sampling dates carry the majority of the information regarding the reproductive number. Finally, we provide genome-based estimates of the cumulative number of infections for each outbreak. For 7 out of 11 of the populations studied, the number of confirmed cases is much bigger than the cumulative number of infections estimated from the sequence data, a possible explanation being the presence of unsequenced outbreaks in these populations.

## Significance

At the beginning of the COVID-19 outbreak, researchers around the globe estimated the rate at which the disease spread through populations prior to public health intervention, as quantified by the parameter $R_{0}$. This quantity is often estimated based on case count data and may be biased due to the presence of import cases, which may give the appearance of elevated local transmission. To overcome this, we estimated $R_{0}$ by applying Bayesian phylodynamic methods to SARS-CoV-2 genomes. Here, we provide $R_{0}$ and absolute infection count estimates for 15 distinct outbreaks. Some of these presented estimates already contributed to our understanding of the baseline transmission dynamics of the disease in early 2020 prior to cases of COVID-19 having appeared in many countries.

Severe acute respiratory syndrome coronavirus 2 (SARS-CoV-2) and the corresponding disease COVID-19 spread rapidly around the globe. By the end of the first year of the pandemic, over 65 million confirmed cases and over 1.5 million deaths had been reported globally (1).

In early 2020, it was necessary to accurately quantify the underlying transmission dynamics of the virus and, in particular, its basic reproductive number (2) in order to understand the global threat this pandemic was to pose. Such information was used to determine the likely future trajectories of individual outbreaks. Inference of pathogen transmission dynamics is traditionally achieved using either line lists (3) composed of case confirmation times, locations and patient details, or aggregated incidence reports, which are typically updated daily. This approach was being widely applied (4–8) by various groups around the world seeking to both understand the early pandemic spread and, later on, to monitor transmission dynamics in real time. Platforms such as EpiForecasts (9), or our own (10), reported frequently updated estimates of the reproductive number throughout the pandemic.

Despite the wide-spread application of such methods, the estimates produced from incidence data (line lists or aggregated reports) alone are inherently susceptible to several biases and limitations (11–14). First, the presence of pools of undiagnosed infected individuals, together with changes in testing methods and the extent to which testing is happening at all, can lead to misleading characterizations of the epidemic. While this can be mitigated to some extent through the use of hospitalization or mortality data in place of positive test data, such data may not be available to early-outbreak studies due to data privacy considerations. Second, while line lists may sometimes contain information on the infection locations, in the majority of cases, it is impossible to discriminate between import cases and those attributable to local transmission based on incidence reports. This has the potential to produce overestimates of local transmission rates. Estimating rates and directions of transmission between geographic regions is similarly impeded. Third, on their own, these line list data do not provide information about the state of outbreaks before the first confirmed case.

Characterizing transmission dynamics is critical to the successful design of public health interventions. Thus, finding ways around potential biases and limitations when quantifying transmission dynamics is crucial. Early testing efforts have been paralleled by significant efforts to sequence SARS-CoV-2 genomes from the initial outbreak andsubsequent pandemic in “real time.” Many of the groups responsible for sequencing SARS-CoV-2 genomes generously chose to make them available immediately to the research community via the GISAID platform (15). These data were used successfully for the development of testing assays (16) and for learning about the molecular structure of the virus (17, 18). Importantly, the continued and widespread sequencing efforts also enabled—in combination with phylodynamic methods (19, 20)—independent, and potentially more robust, estimates of very early transmission dynamics.

Phylodynamic methods couple epidemiological models with models of sequence evolution, allowing us to estimate transmission dynamics based on the relationships between SARS-CoV-2 genome sequences. Several early studies made use of SARS-CoV-2 sequence data in a phylodynamic context to investigate early pandemic spread. For example, Lai et al. (21) inferred early dynamics of the global effective reproductive number, using all available sequences at the date of publishing, obtaining an $R_{0}$ estimate of 2.6, with a 95% highest posterior density (HPD) interval of [2.1,5.1]. In contrast, Geidelberg et al. (22) focused on a specific Weifang-associated outbreak cluster and used a compartment model to infer a basic reproductive number of 3.4, with a 95% credible interval [2.1,5.2]. Furthermore, a phylodynamic study of the early infection dynamics within four island countries (New Zealand, Australia, Iceland, and Taiwan) by Douglas et al. (23) used a geographic compartmental model to infer lower reproductive numbers, e.g., 1.41 (95% HPD 1.07,1.89) for New Zealand, while Danesh et al. (24) used a single-compartment phylodynamic model to infer an early reproductive number in France of 2.56 (95% HPD [1.66,4.74]). Genomes have also been coupled with extremely detailed agent-based models to infer the probable sources of infection for specific COVID-19 cases within the Australian population (25). A comprehensive review of these and other phylogenetic and phylodynamic approaches which were applied during the early phase of the pandemic has been assembled by Attwood et al. (26).

In this paper, we report on an early study whose goal was to infer the basic reproductive number ($R_{0}$) for each of 15 distinct outbreaks distributed among 11 populations (10 countries and the Diamond Princess cruise ship) by applying Bayesian phylodynamic methods to genomes collected between January and March of 2020. While other genomes from the same period have since become available, we focus only on data which were made available shortly after the start of the pandemic to showcase what kind of early findings were feasible. Importantly, these genomes are not only used as the basis for the phylodynamic analyses but also in the identification of probable country-specific transmission clusters. We apply a statistical test to determine whether $R_{0}$ differs by outbreak, or among certain sets of outbreaks. Finally, we provide Bayesian estimates of cumulative case counts over time for each of the outbreaks, illustrating possible trajectories each outbreak took even before the first samples were collected. The $R_{0}$ estimates for some of these outbreaks were made available by us on the “Virological” forum in 25 February 2020, prior to widespread mitigation measures https://virological.org/t/evolutionary-epidemiological-analysis-of-93-genomes/405.

## Results

We used the Nextstrain (27) platform to identify clusters of SARS-CoV-2 genome sequences likely sampled from individuals within the same outbreak and selected only those samples collected prior to or just after the introduction of strong public health interventions in the associated locations. Importantly, while we used samples from a particular country if available (France, Iceland, the Netherlands, Spain, Wales, and Washington State, USA), we also included sequences from cases that were exposed in the region of interest and subsequently traveled abroad (i.e., a travel sentinel) for countries where no or few sequences were available or sequencing seemed very biased (Italy, Iran, and China before the quarantine of Wuhan). As expected, these outbreaks show high rates of sequence identity within cluster samples, but almost never between samples (*SI Appendix*, Fig. S1).

The Diamond Princess outbreak is an exception to this protocol, as the interventions were put in place immediately on the date corresponding to the first sequenced sample. We include it despite this complication because, as a well-studied outbreak in a relatively isolated population, it provides useful validation of our inference methods. (Refer to the *Materials and Methods* for the full details of the sample selection procedure for all outbreaks.)

We then applied the Bayesian phylodynamic framework BDSKY (19), to co-infer $R_{0}$ along with the probability of an infected person being included in our dataset, and the underlying viral phylogenetic tree for each cluster. In our context, this framework assumes that each outbreak was produced by an independent birth–death process parameterized by a reproductive number (the ratio of birth rate to death rate), a “become uninfectious” rate (the death rate), and a sampling proportion (the probability of an infectious individual testing positive and their sample being subsequently sequenced). Both sample times and the times of ancestral birth events on the transmission tree are sources of signal for the final birth–death inference results. Inference was done under the assumption of constant transmission (birth) rates for each cluster, with the sole exception of the Diamond Princess, where we allowed for the transmission rate to shift at the time of the onboard quarantine. (Refer to the Phylodynamic analyses portion of the *Materials and Methods* for full details of this analysis.)

**Fig. 1.**
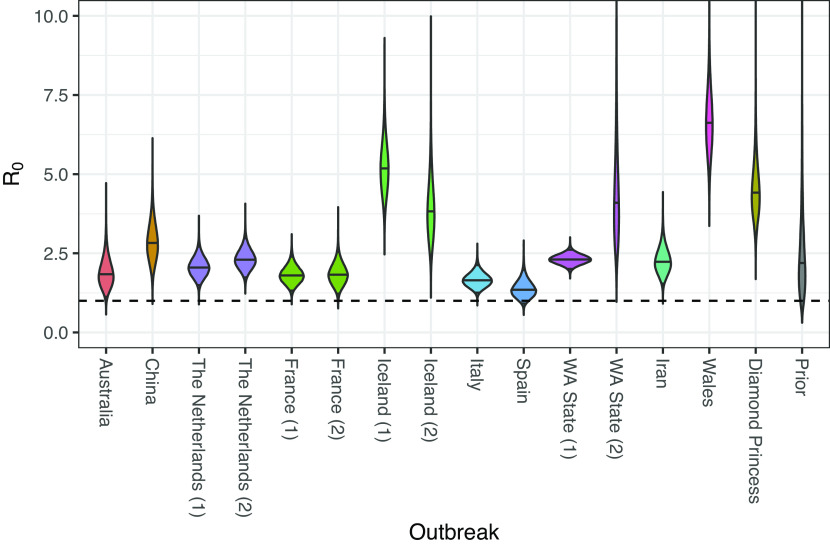
Posterior distributions for reproductive numbers for outbreaks considered in this study. Solid horizontal lines represent median values; the dashed horizontal line represents the threshold between exponential growth and decline of an outbreak.

Fig. 1 illustrates the posterior distributions for $R_{0}$ inferred for each of the outbreaks, together with the prior distribution for comparison. Interestingly, rather than a continuum of values, our analysis seems to isolate several distinct modes. The median posteriors for the majority of outbreaks lie between 1.4 and 2.9. However, the $R_{0}$ values inferred for the two outbreaks associated with Iceland, the Welsh outbreak, a Washington State (USA) outbreak, and the Diamond Princess outbreak have posterior median values ranging between 4 and 7.

We went on to investigate the statistical support for groups of outbreaks indeed sharing the same $R_{0}$ value. To do this, we used a Bayesian model averaging (28) scheme (described in the Phylodynamics analyses portion of the *Materials and Methods*) in which different groupings of outbreaks sharing $R_{0}$ values were proposed as different models. This identified support for many unique $R_{0}$ values among the 15 outbreaks (median 9 unique values, central 95% credible interval [3,15]; see *SI Appendix*, Fig. S2 for the posterior distribution). The corresponding posterior distributions for the outbreak-specific $R_{0}$ values generated by this analysis are shown in *SI Appendix*, Fig. S3.

A comparison of the pre- and post-quarantine effective reproductive number estimates for the Diamond Princess outbreak is shown in *SI Appendix*, Fig. S4, and shows a significant drop in transmission rate following the implementation of isolation measures.

The sampling proportion (proportion of infected individuals sampled for sequencing) in each outbreak was also inferred as part of this analysis and these results are shown in *SI Appendix*, Fig. S5. Note that many of the sampling proportion median estimates are very high (on the order of 50% or higher). In interpreting these values, it is very important to consider that these parameterize the outbreak specific to the sequences sampled, which likely only involve a small subset of the cases belonging to a particular region.

As mentioned already, birth–death phylodynamic results are dependent not only on the genomic data but also on the distribution of sample collection dates. In fact, we find that in our analyses, the sample collection dates carry most of the information regarding $R_{0}$. We demonstrated this by running an additional set of “date only” phylodynamic analyses in which the genomic sequences were treated as unknown. Additionally, we applied both a simplistic linear regression approach (*Materials and Methods*) and an established traditional approach (13) to the cumulative sequence counts. The results of these alternative analyses are summarized in *SI Appendix*, Fig. S6 and—in many cases—show relatively close agreement, albeit with slightly less certainty in the phylodynamic estimates, compared to those shown in Fig. 1. We also ran 10 additional analyses in which the association between the sequences and the sampling dates was randomized within each cluster. The marginal $R_{0}$ posteriors from each of these “shuffled” analyses are shown in *SI Appendix*, Fig. S7 alongside the corresponding estimates from the original (unshuffled) analyses and those of the sequence-free analyses, where it is clear that permutation of these associations has very little impact on the inference results. (A small remaining difference between the estimates from the shuffled analyses and those from the dates only analyses indicates that the diversity within the cluster-specific sequences is still sufficient to weakly inform the age of the outbreak).

Given this dominating effect of the sampling times, it is natural to consider how sensitive our results are to the assumption that the sampling rate and reproductive number are fixed over the time period of each outbreak. We thus performed a separate set of analyses in which these quantities were allowed to change at a point at the center of the sampling window of each outbreak (excluding the Diamond Princess outbreak). The resulting $R_{0}$ estimates, presented in *SI Appendix*, Fig. S8, show no major change in the results compared with those in Fig. 1, with the exception of the Netherlands (1) and WA State (1) outbreaks which suggest higher $R_{0}$ values. In addition, direct comparison between the cumulative sequence and confirmed case count distributions (*SI Appendix*, Fig. S9) confirms that in most cases, the temporal distribution of included sequences is comparable to the distribution of confirmed cases, further supporting our assumption of constant sampling rate within this interval.

In order to investigate how much our results are affected by the prior, we repeated the fixed-rate analyses with a broader prior on $R_{0}$. This broad prior did not qualitatively change the results compared to our main analysis (*SI Appendix*, Fig. S10). That said, the broader prior did increase the magnitude of $R_{0}$ estimates associated with both the second WA State outbreak and the Welsh outbreak, suggesting that the combination of genetic data and sample times less strongly inform these parameters than those for the other outbreaks.

The BDSKY framework applied above marginalizes over the outbreak-specific case count trajectories that are nonetheless an integral part of the underlying birth–death model. Using a recently developed particle filter approach (29), we were able to additionally impute these trajectories and hence sample the posterior distribution for the cumulative trajectories of the number of infections corresponding to each outbreak. In the cases where two outbreaks are associated with the same location, the inferred number of infections are combined. (Additional details are given in the Phylodynamic analyses portion of the *Materials and Methods*.)

**Fig. 2.**
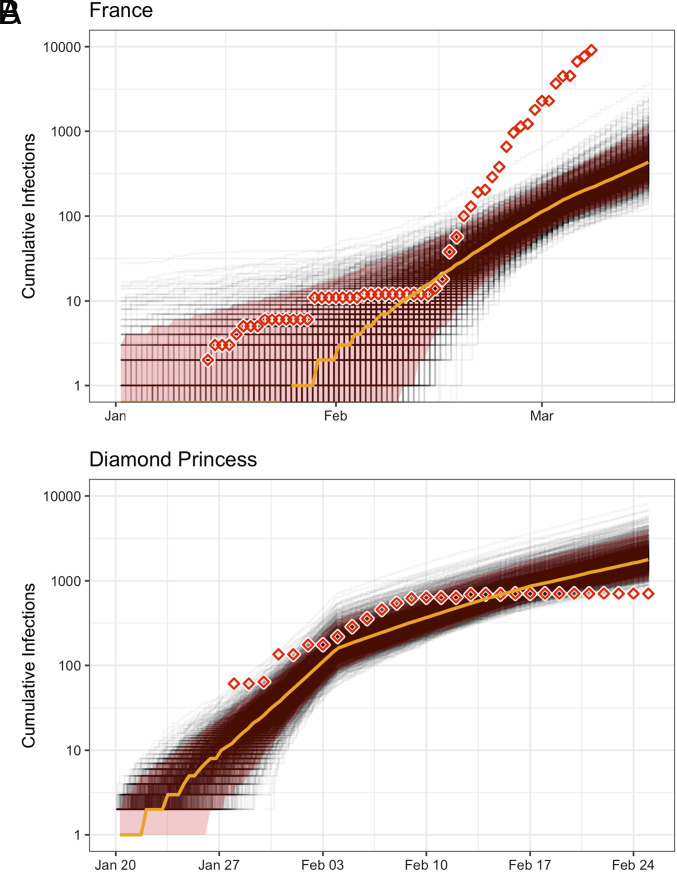
Inferred cumulative number of infections through time for (*A*) France, and (*B*) the Diamond Princess cruise ship. Gray lines indicate individual trajectories sampled from the posterior, the orange line indicates the posterior median, and the red shaded area indicates the 95% central posterior density interval for the number of infections at each point in time. Inferred numbers of infections are shown together with the corresponding confirmed case counts (diamonds) in each population as recorded by Dong et al. (30), which are offset by 10 d to account for the delay between infection and case confirmation (8). Note that these inferences concern only those infections associated with the specific outbreak from which the sequence data are drawn, as detailed in the discussion section. The total number of infections may have been much higher (Inference for remaining outbreaks are shown in *SI Appendix*, Fig. S11).

**Fig. 3.**
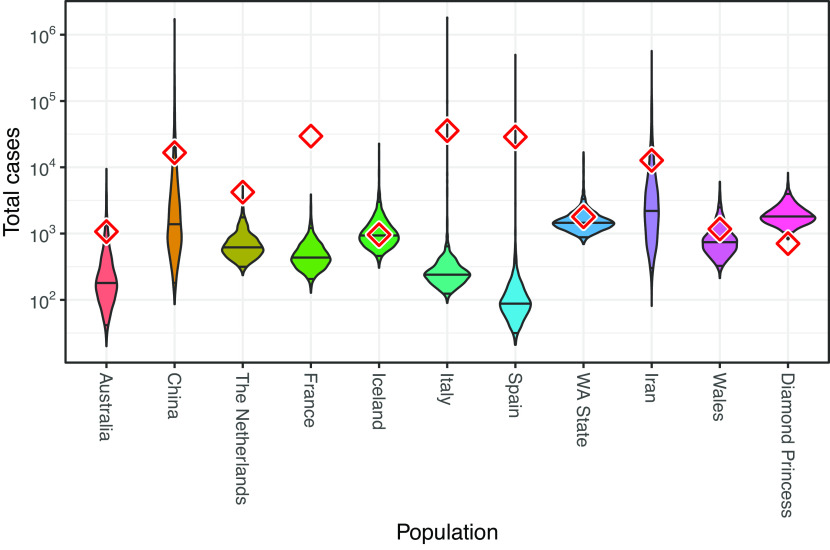
Estimates of the total number of infections estimated from phylodynamic analyses, with diamonds indicating confirmed case counts obtained from ref. 30, offset by 10 d to account for the delay between infection and case confirmation (8). The numbers are for the date of the final genome sample considered in each population. We note that we have likely analyzed only a subset of the total number of outbreaks which were circulating in each country.

Inferred cumulative trajectories of the number of infections for the French and Diamond Princess outbreaks are shown in Fig. 2 alongside the daily number of confirmed cases as reported by the Center for Systems Science and Engineering at Johns Hopkins University (30), to which we have applied a 10-d offset in order to account for the estimated delay between infection and case confirmation (8). Similar case count trajectories for the remaining populations are provided in *SI Appendix*, Fig. S11. The posterior distributions for case counts at the time of the most recent genome sample are shown for all populations in Fig. 3. In several instances (e.g., China, WA State, and the Diamond Princess) the inferred case counts are comparable to the number of confirmed cases. However, in many instances, they differ quite dramatically. As with the sampling proportion estimates, interpretation of the inferred number of infections must be made very carefully, in the knowledge that these estimates only correspond to infections associated with the specific outbreaks for which we have sequence data. On one hand, the outbreaks represented by the sequences included in our study may represent only a subset of the outbreaks actually active in a particular population/country. If this is the case, inferred infection counts would only include infections belonging to those sampled outbreaks and may thus underestimate the true cumulative number of cases in the population. On the other hand, outbreaks may also involve ancestral cases which lay outside the region of interest, meaning the estimated number of infections may actually be higher than the true regional number of infections. With our results, it seems that unsampled sequence diversity is likely the dominant effect.

This hypothesis is also supported by a comparison with rough estimates of the total case count extrapolated from population-specific death statistics using the infection fatality ratio (*SI Appendix*, Figs. S12 and S13). This comparison suggests that for Iceland and the Diamond Princess, we may have sequences for all outbreaks having occurred during the considered time interval. For the other regions, many outbreaks may not have been sequenced.

### Discussion.

Our central result is that prior to strong public health interventions, the majority (10) of the outbreaks studied seem to have grown at rates with median $R_{0}$ values ranging between 1.4 (Spain) and 2.8 (China).

The specific case of the Diamond Princess is interesting, as the details of this outbreak are well known and, at least for the time period affecting our analysis, the population involved was strictly isolated (i.e., we can say with a high degree of certainty that no immigration or emigration occurred). In this case, we believe the high pre-intervention $R_{0}$ estimate reflects a real elevated infection rate caused by unchecked transmission within the relatively confined on-board environment.

The remaining outbreaks to which higher $R_{0}$ values are attributed are limited to those with the shortest sampling windows (*SI Appendix*, Fig. S14). Given the strong role played by sample times in these inferences, it is therefore possible that these values are the result of bias due to sampling model misspecification and that this problem is exacerbated by the short sampling windows involved. The sampling model used for these outbreaks assumes that each infected individual is sampled with a constant, outbreak-specific, probability: the sampling proportion. We showed that allowing for a single shift in both the sampling proportion and $R_{0}$ during the outbreak did not result in much lower $R_{0}$ values for these remaining outbreaks, but this still assumes that sample times occur at a rate linearly proportional to the number of active cases within the smaller intervals. Additionally, the noted prior dependence of the $R_{0}$ values for the second WA State outbreak and the Welsh outbreak suggests that these estimates in particular are less robust than those of the other outbreaks.

Another potential source of upward bias on $R_{0}$ is the process of outbreak selection. We necessarily restrict our attention to outbreaks for which sufficient data exist to provide statistical signal. This restriction may have the effect of selecting for steeper outbreak trajectories. Since the birth–death models under which we perform the inference do not account for this conditioning, these steeper trajectories will be interpreted as evidence for larger $R_{0}$, even when the increased gradient is simply the result of demographic noise in the growth of the epidemic. Including appropriate conditioning in phylodynamic inference to guard against this kind of bias will be the focus of future research.

Given that most of information content in the genome sequence data analyzed seems to come from sampling times, it is natural to wonder whether the phylodynamic approach truly does offer additional insights into these outbreaks, beyond those offered by traditional incidence data. After all, the temporal distribution of our genomic samples is subject to many of the same testing trends and biases that affect incidence data, despite the care taken to exclude non-randomly sampled sequences. Our primary answer to this question is that, genomic data allowed us to assign samples to region-specific outbreaks (excluding travel cases, for instance) in the absence of contact tracing data, which is often not available for study. This was possible because the genomes contained sufficient phylogenetic signal to identify outbreak-scale clusters, despite lacking sufficient signal to contribute significantly to the within-outbreak phylodynamic parameter estimates. Furthermore, even though the impact of the phylogeny within each identified outbreak on the inferred epidemic parameters was negligible, the application of phylodynamic methods yields information about the total number of outbreak-specific infections through time, including those infections which have gone undetected.

There is also a chance that the outbreak identification was imperfect, meaning that some clusters may have resulted from more than one introduction event. This would mean that the corresponding $R_{0}$ values are indicative not only of the transmission dynamics of the study population but also of the populations from which the introductions occurred. However, the majority of branching events in the inferred tree should be within-outbreak transmissions, and only few ancestral branching events should be introductions, meaning the bias should not be a dominating signal. One way in which this could be addressed formally would be to involve so-called “multi-type” birth–death models (31) that allow for uncertainty in the ancestral population membership of transmission tree lineages.

We emphasize again that extreme care must be taken when interpreting both the inferred number of total infections and the clinically confirmed case counts. First, our inferences correspond to the number of infections associated only with the specific outbreaks from which the genomic data originate. It is highly likely that additional outbreaks, from which we do not have genetic data, occurred within a given population during the time periods considered. The large inferred sampling proportions support this hypothesis, as do the comparison with case counts extrapolated from population-specific death statistics. Such cryptic outbreaks could contribute to the confirmed case counts but would be absent from our phylodynamic inference. Second, the confirmed case numbers themselves can only provide a lower bound on the true number of cases in a population. Taken together, these points imply that the larger of the phylodynamically inferred number of infections and the corresponding confirmed case counts provides a lower bound on the true number of cases within each population. (As outlined in the *Results*, one could also think of reasons that the phylodynamic estimates are overestimates, but our data do not indicate such biases to be present).

We highlight in this paper the importance of SARS-CoV-2 genomes for quantifying transmission dynamics. In particular, we provide estimates for the basic reproductive number which are complementary to classic epidemiological studies. Our phylodynamic analyses of SARS-CoV-2 genomes confirm the $R_{0}$ estimates for Wuhan (5) and provide estimates for 15 outbreaks across 11 populations around the world for which classic epidemiological methods may be problematic due to the difficulty of disentangling introductions from local transmissions. Even when within-outbreak genomic diversity (and thus sequence-based phylodynamic signal) is low, genomes aid epidemiological understanding by allowing the identification of independent local transmission clusters, and thus avoiding biases due to recently introduced cases from the outside. While details vary between countries, our estimates are in rough agreement with more classical non-genomic estimates of the reproductive number from platforms such as EpiForecasts (9) and that described by Huisman et al. (10), which indicate that the median estimates for the basic reproductive number for the populations studied here lie between 1.5 and 3. Going forward, we envision that genomes will become an integral part of epidemiological and pandemic assessment. Indeed, for patients whose infection is not traceable, it is the genomes which contain valuable information for linking them into the transmission chain and thus quantify transmission dynamics.

## Materials and Methods

### Outbreak Identification and Sample Selection.

The birth–death models we employ assume that genome samples are taken uniformly at random from the infectious population for a short time during the early, exponential growth phase of each outbreak. Since our analysis is necessarily retrospective rather than prospective, we devised two strategies to approximate such a sampling scheme using publicly available samples from GISAID (15). For sparsely sampled, unsampled, or clearly non-uniformly sampled outbreaks (Italy, Iran, and China before the quarantine of Wuhan, respectively), we included sequences from cases that were exposed in the region of interest and subsequently traveled abroad, where they were then diagnosed and sampled. The sequences attributed to the Iranian outbreak, for example, are all travel cases isolated and sequenced in Australia (32). For more densely sampled outbreaks (France, Iceland, the Netherlands, Spain, Wales, and Washington State, USA), we analyzed samples that were exposed and sampled within the region of interest. For these outbreaks, we considered only samples that clustered together with other samples from the same region in a phylogenetic tree of the global pandemic (27). This was done in order to sample primarily within-region transmission events.

#### Sample acquisition and curation.

We downloaded all sequences available on GISAID (15) on 1 April 2020. After quality-filtering this sequence set, we aligned the sequences, built a phylogenetic tree, and identified regional outbreak clusters within the tree. Sequence quality control, alignment, and tree building were all performed using the Nextstrain pipeline adapted to SARS-CoV-2 (33).

We first filtered the available sequences to exclude sequences shorter than 25,000 base pairs, sequences with imprecise sampling dates, known re-samples of the same case, low-quality sequences (as determined by Nextstrain), and all but one sequence from known epidemiologically linked cases. We note that our knowledge of which samples come from epidemiologically linked cases (as identified by Nextstrain and gleaned from media reports) is far from exhaustive. Whenever we were able to access this information, we used it to exclude non-randomly sampled sequences, but in many cases, the relevant information was either not collected or not readily accessible.

#### Alignment and outbreak detection.

After these filtering steps, we aligned the remaining sequences to a reference genome generated from an early COVID-19 patient in Wuhan (GenBank accession number MN908947) (34). SNPs in the first 130 sites, last 50 sites, and at sites 18529, 29849, 29851, and 29853 were masked from the alignment because they are likely sequencing artifacts (33).

We built a maximum-likelihood phylogenetic tree with IQ-TREE (35) using this alignment. We then picked clades from this tree where sufficient (≥ 9) samples from the same region clustered together. We assume that these clusters represent primarily within-country transmission events rather than introductions from abroad.

Exceptionally for the Italy, Iran, and China outbreaks, we additionally identified samples from cases that were presumably exposed to the virus in these regions but were sampled abroad (travel cases). The dataset for Italy included sequences from both non-travel and travel cases, while those for China and Iran were composed exclusively of sequences from travel cases. This exposure information comes from metadata available on GISAID and Nextstrain as well as information provided by sequencing centers and in media accounts.

#### Sample set truncation.

To limit sampling to the early, exponential growth phase of each regional outbreak, we truncated sampling based on the dates of major public health interventions (*SI Appendix*, Table S1). We retained only samples collected before or on the date of these public health interventions, with the exception of the Iran, Iceland, and Spain outbreaks. For these outbreaks, we extended the time cutoff so that the sample size was not prohibitively small. (The extension for Iran was 11 d, for Iceland, it was 2 d, and the cutoff for Spain was extended by 1 d, as shown in *SI Appendix*, Table S1.) Since the transmission events leading to sampled cases happened at least a few days before sampling, these cutoffs should, for the most part, be conservative.

### Bayesian Phylodynamic Analyses.

We use the BDSKY package (19) of BEAST 2 (36) to perform Bayesian phylodynamic inference of outbreak-specific basic reproductive numbers and sampling proportions from the sequence alignments. This approach employs a Markov chain Monte Carlo (MCMC) algorithm to produce samples from the joint posterior distribution of all outbreak-specific phylogenetic trees and model parameters, conditional on the available sequence data.

All analyses described below were repeated five times, each with different pseudo-random number generator seeds. These replicates were then compared to assess convergence, then combined. In all cases, the effective sample size of all sampled parameters exceeded the usual threshold of 200 commonly used as a MCMC quality threshold in the phylodynamics literature. Additionally, we used traces of the topological path distance (37), as implemented in the “R We There Yet” package (38), to assess the quality of the tree space component of the sampling. (For example, *SI Appendix*, Fig. S15 compares visualization of the outbreak-specific tree space distributions sampled by two of the five replicates generated as part of the first analysis described below and indicates excellent tree space sampling).

#### Main analysis.

Our primary analysis involved using MCMC to characterize the following joint posterior distribution:

$$\begin{array}{cc} P({\vec{R}}_{0},\vec{s},\vec{T}|\vec{A},\mu ,b)= & \frac{1}{P(\vec{A}|\mu ,b)}\sum_{{\vec{t}}_{\mathrm{or}},\kappa ,\gamma }\prod_{c}[P_{\;\text{HKY}+\Gamma }\left(A^{\left(c\right)}|T^{\left(c\right)},\mu ,\kappa ,\gamma \right)\\ & \times P_{\;\text{BDSKY}}\left(T^{\left(c\right)}|R_{0}^{\left(c\right)},s^{\left(c\right)},b\right)P\left(s^{\left(c\right)}\right)P\left(R_{0}^{\left(c\right)}\right)]\\ & \times P\left(R_{e}^{\mathrm{DP}}\right)P\left(\kappa \right)P\left(\gamma \right). \end{array}$$

For clarity, all parameters in this analysis are described together with their priors (or chosen values, where appropriate) in Table 1.

**Table 1.** Explanation of notation used in the description of the mathematical model, together with priors (in the case of estimated parameters) and values (in the case of fixed parameters) used in the main analysis

Notation	Definition	Prior or value (main analysis)
$R_{0}^{\left(c\right)}$	Basic reproductive number for outbreak c	LogN(0.8,0.5)
$R_{e}^{\mathrm{DP}}$	Post-quarantine effective reproductive number for Diamond Princess outbreak	LogN(0.8,0.5)
$s^{\left(c\right)}$	Case sequencing probability for outbreak c	Beta(1,4)
b	Become-uninfectious rate (per year)	36.5
$t_{\mathrm{or}}^{\left(c\right)}$	Time of origin for outbreak c in years	LogN(−2,0.8)
$A^{\left(c\right)}$	SARS-CoV-2 genome alignment for outbreak c	–
$T^{\left(c\right)}$	Phylogenetic tree for outbreak c	BDSKY (19)
μ	SARS-CoV-2 substitution rate (per site per year)	$8\times 10^{-4}$
κ	Transition-transversion substitution rate ratio of HKY model	LogN(1,1.25)
γ	Shape parameter for Γ-distributed site-site rate variation	Exp(0.5)

Sequence alignments were analyzed jointly as part of a Bayesian phylodynamic analysis using the BDSKY package (19) of BEAST 2 (36), using a single HKY substitution model (39) allowing for Γ-distributed site-site rate variation (40) with a strict clock rate μ fixed to $8\times 10^{-4}$ substitutions/site/y following Nextstrain (27). The tree $T^{\left(c\right)}$ corresponding to each outbreak cluster c was assumed to be produced by a birth–death process with reproductive number $R_{0}^{\left(c\right)}$, sampling proportion $s^{\left(c\right)}$ and become-uninfectious rate b. In each case, the sampling proportion for the outbreak was assumed to be zero before the first included sample for that outbreak. In the special case of the Diamond Princess outbreak, a second (effective) $R_{0}$ value, $R_{e}^{\mathrm{DP}}$ was associated with the days following the on-board intervention. All $R_{0}$ values and $R_{e}^{\mathrm{DP}}$ were assumed to be independent and given a LogNormal(0.8,0.5) prior. The time between the start of the birth–death process associated with each outbreak and the time of the most recent sample for the same outbreak was given a LogNormal(−2,0.8) prior. The value of the become uninfectious rate b was fixed to 36.5, equivalent to an expected time until becoming uninfectious for each individual of 10 d. This is in line with the estimates of the latent and infectious periods provided by Li et al. (4) and follows the assumptions used by Scire et al. (8). The prior for each the sampling proportion was chosen to be Beta(1,4), which prioritizes low sampling probabilities without completely excluding higher probabilities.

The variables and priors used for this analysis are summarized in Table 1.

#### Bayesian model averaging analyses.

A second analysis was run with an identical model configuration to the first analysis, aside from its use of Bayesian model averaging to quantify the number of unique $R_{0}^{\left(c\right)}$ values needed to describe the outbreaks. This was done by replacing the original priors over the cluster-specific $R_{0}$ values with a single Dirichlet process prior (DPP) applied to the vector ${\vec{R}}_{0}=\left[R_{0}^{\left(1\right)},R_{0}^{\left(2\right)},\ldots ,R_{0}^{\left(15\right)}\right]$:

[1] $$\begin{array}{c} P\left({\vec{R}}_{0}\right)=H\left(R_{0}^{\left(1\right)}\right)\prod_{c=2}^{15}q\left(R_{0}^{\left(c\right)}|R_{0}^{\left(1\right)},\ldots ,R_{0}^{\left(c-1\right)}\right), \end{array}$$

where

[2] $$\begin{array}{cc} q(R_{0}^{\left(c\right)}|R_{0}^{\left(1\right)},\ldots ,R_{0}^{\left(c-1\right)})= & \frac{\alpha }{c-1+\alpha }H\left(R_{0}^{\left(c\right)}\right)\\ & +\frac{1}{c-1+\alpha }\sum_{i=1}^{c-1}\delta \left(R_{0}^{\left(c\right)}-R_{0}^{\left(i\right)}\right), \end{array}$$

and δ(·) represents the Dirac delta function. Here, H and α are the base distribution and intensity parameter of the DPP, respectively. We set the base distribution to LogN(0.8,0.5), the same prior used for the $R_{0}^{\left(c\right)}$ components in the main analysis. We implement the DPP using a reversible jump Markov chain Monte Carlo algorithm, as described in *SI Appendix*, Text. Following the prescription of ref. 41, we applied a Gamma hyperprior Γ(0.512,0.029) to the intensity parameter such that the marginal prior distribution for the number of unique elements of ${\vec{R}}_{0}$ was approximately uniformly distributed between 1 and 15.

#### Sensitivity analyses.

We ran two additional analyses to determine the sensitivity of our conclusions to the model assumptions. First, to test the robustness with respect to changes in the $R_{0}$ priors, we ran a separate analysis using a Unif(0,10) prior for each $R_{0}^{\left(c\right)}$ parameter. Second, we ran an analysis in which both $R_{0}^{\left(c\right)}$ and $s^{\left(c\right)}$ were allowed to change once during each outbreak, at a time midway between the first and last sample assigned to that outbreak.

#### Sample-date only and shuffled sequence analyses.

In order to assess the relative impact of the sequence data on these $R_{0}^{\left(c\right)}$ estimates, another joint phylodynamic analysis was performed using the same setup as the first, but without any sequence data.

Additionally, for comparison, a (overly) simple regression inference of the $R_{0}^{\left(c\right)}$ was conducted by assuming that the number of active infections associated with each outbreak grew according to the deterministic function $N^{\left(c\right)}\left(t\right)=\;\exp\left[b\left(R_{0}^{\left(c\right)}-1\right)t\right]$. This implies that the logarithm of the cumulative number of samples grows linearly at the rate $b(R_{0}^{\left(c\right)}-1)$, which we then fit to the empirical cumulative sample numbers from each outbreak.

In order to test the robustness of the phylodynamic estimates of the outbreak-specific $R_{0}^{\left(c\right)}$ values, we applied EpiEstim (13) to the same sample time distributions used for the regression analysis. In these analyses, $R_{0}^{\left(c\right)}$ was assumed to be constant through time in each outbreak. A serial interval of mean 4.8 d and a SD 2.3 d was used (42).

Finally, to assess the degree to which the actual association between individual sequences and sampling dates was useful for cluster-specific $R_{0}^{\left(c\right)}$ estimates, we performed 10 additional joint phylodynamic analyses of “shuffled” alignments where this association was randomized within each cluster.

#### Case count trajectory inference.

Inference of cumulative case count trajectories was achieved by applying the particle filter algorithm implemented in EpiInf (29) to the outbreak-specific tree and parameter posteriors produced by the corresponding BDSKY analyses. This particle filter algorithm can produce trajectories of cumulative case load from the posterior distribution of such trajectories conditional on a given transmission tree and set of birth–death model parameters (in our case, $R_{0}^{\left(c\right)}$, $s^{\left(c\right)}$, and b). Applying this sampling approach to each of the trees and trajectories sampled during a BDSKY analysis thus produces trajectories sampled from the posterior distribution of such trajectories conditional on the same data and priors provided to the original BDSKY analysis. To produce the trajectory posteriors presented in this manuscript, we applied this trajectory sampling approach to each of the outbreak-specific tree and parameter posteriors produced by the first BDSKY analysis described in *Main Analyses* above.

### Estimates of the Number of Infections.

In order to gain an indication of the number of COVID-19 outbreaks not having been sequenced, we compared our phylodynamic case count estimates with estimates imputed by scaling available country-specific death statistics (30) by the inverse of a published estimate (43) of the infection-fatality ratio (IFR) of 0.64% [95% credible interval (0.38%,0.98%)]. For comparison with the phylodynamic case count estimates, we time-shifted the IFR-based case count estimates by −18 d relative to date of death statistics to account for both the elsewhere-assumed 10-d delay between infection and average testing time and a second 8-d delay between positive test results and death (44). We took the ratio between the phylodynamic final cumulative case count estimates and these IFR-based estimates to very approximately represent the fraction of the infections represented in the genomic data.

## Supplementary Material

Appendix 01 (PDF)

## Data Availability

The sequences used in this study were accessed via GISAID (https://gisaid.org) (15). The acknowledgments table available at https://github.com/tgvaughan/R0-manuscript-materials/blob/master/sequences/GISAID_Acknowledgement_Table.csv (45) lists the accession numbers for the sequences associated with each cluster, together with the names of the institutions and authors who generously contributed the sequences. The BEAST 2 XML files used to perform the phylodynamic analyses, together with the R scripts used for post-processing, are available from https://github.com/tgvaughan/R0-manuscript-materials/ (46). All other data are included in the manuscript and/or *SI Appendix*.
