# Supplementary material for: Estimates of early outbreak-specific SARS-CoV-2 epidemiological parameters from genomic data
Source: Proc Natl Acad Sci U S A. 2024 Jan 4;121(2):e2308125121. doi: 10.1073/pnas.2308125121 (PMC10786264; doi:10.1073/pnas.2308125121)
Supplement: Supplementary file 1 — Appendix 01 (PDF) [file pnas.2308125121.sapp.pdf]

Supplementary material for "Estimates of early outbreak-specific SARS-CoV-2 epidemiological parameters from genomic data"

Timothy G. Vaughan, Jérémie Sciré, Sarah A. Nadeau and Tanja Stadler

1 Supplementary Tables and Figures

Table S1: Sequence information.

Outbreak	No. sequences	Date of last sequence	Limiting public health intervention
Australia	9	Mar. 11	Mar. 21 nationwide social distancing begins
China	13	Jan. 23	Jan. 23 Wuhan quarantined
The Netherlands (1)	35	Mar. 12	Mar. 12 schools close, large gatherings banned
The Netherlands (2)	51	Mar. 12	"
France (1)	31	Mar. 16	Mar. 16 nationwide lockdown
France (2)	19	Mar. 16	"
Iceland (1)	47	Mar. 18	Mar. 16 secondary schools close, large gatherings banned
Iceland (2)	17	Mar. 18	"
Italy	55	Mar. 8	Mar. 8 Lombardy lockdown
Spain	14	Mar. 12	Mar. 11 schools close in Madrid
WA State (USA) (1)	217	Mar. 11	Mar. 11 large gatherings banned
WA State (USA) (2)	9	Mar. 11	"
Iran	14	Mar. 4	Feb. 22 schools close, large gatherings banned
Wales	47	Mar. 16	Mar. 20 schools close
Diamond Princess	96	Feb. 25	Feb. 4 ship quarantined

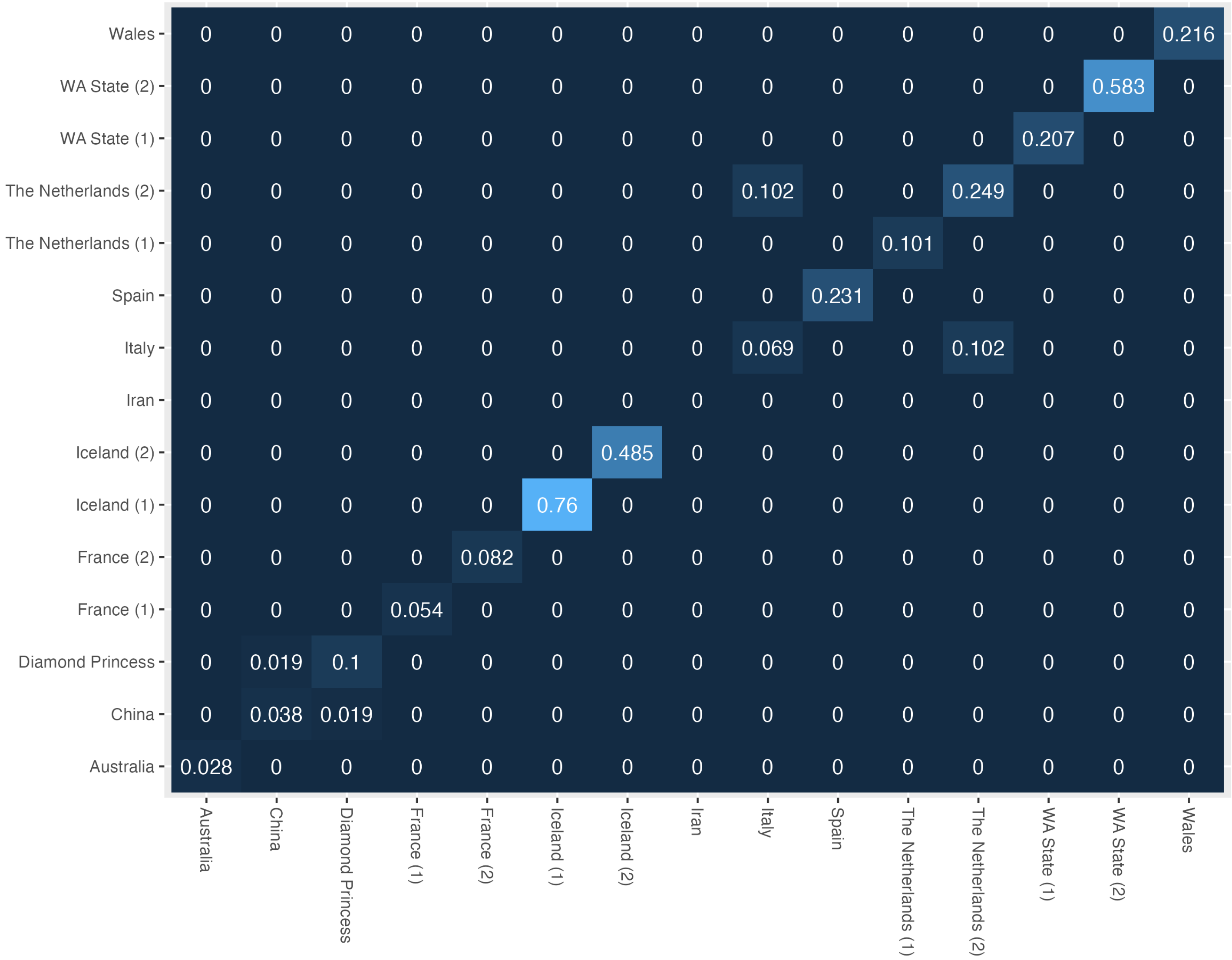

Figure S1: Sequence identity statistics within and between identified outbreak clusters. Each off-diagonal entry in the table corresponds to the fraction of all possible sequence pairs, including one sequence from one cluster and one sequence from another, which are identical. Diagonal entries are similar, but all pairs from a single cluster are considered.

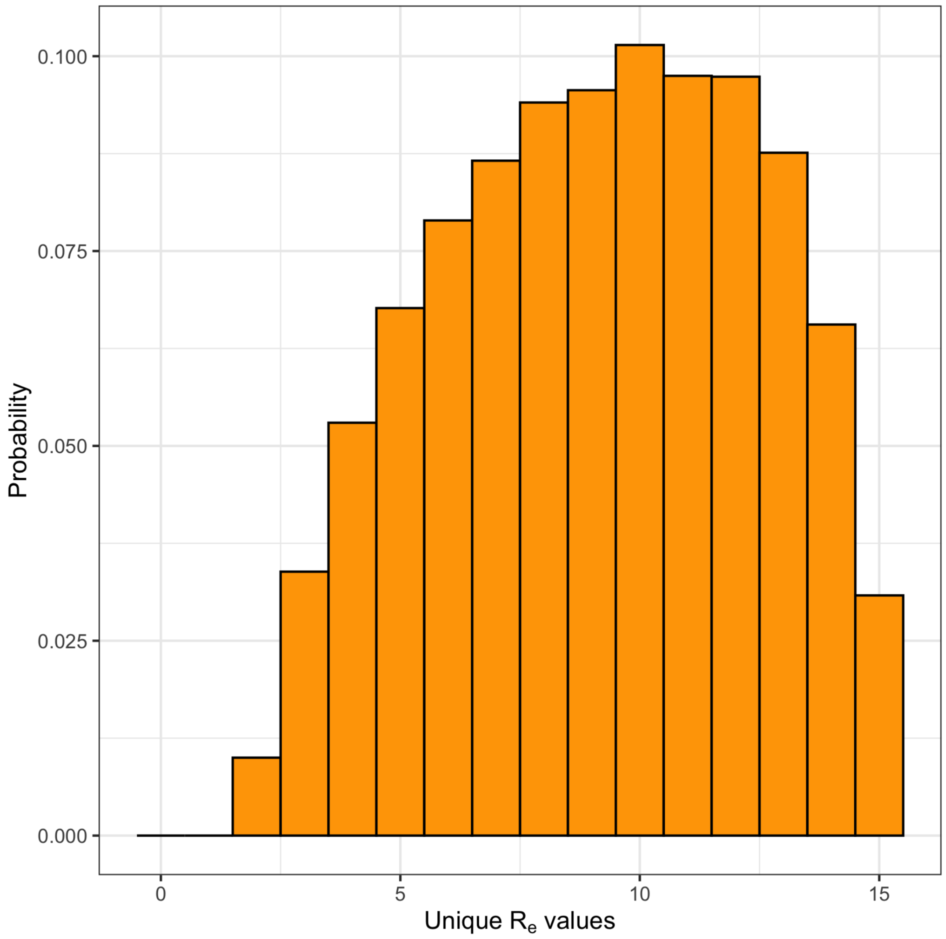

Figure S2: Posterior for the number of unique R_0 values among the 15 distinct outbreaks considered, given by Bayesian model averaging. (Only the value prior to the quarantine aboard the Diamond Princess was included in this averaging.)

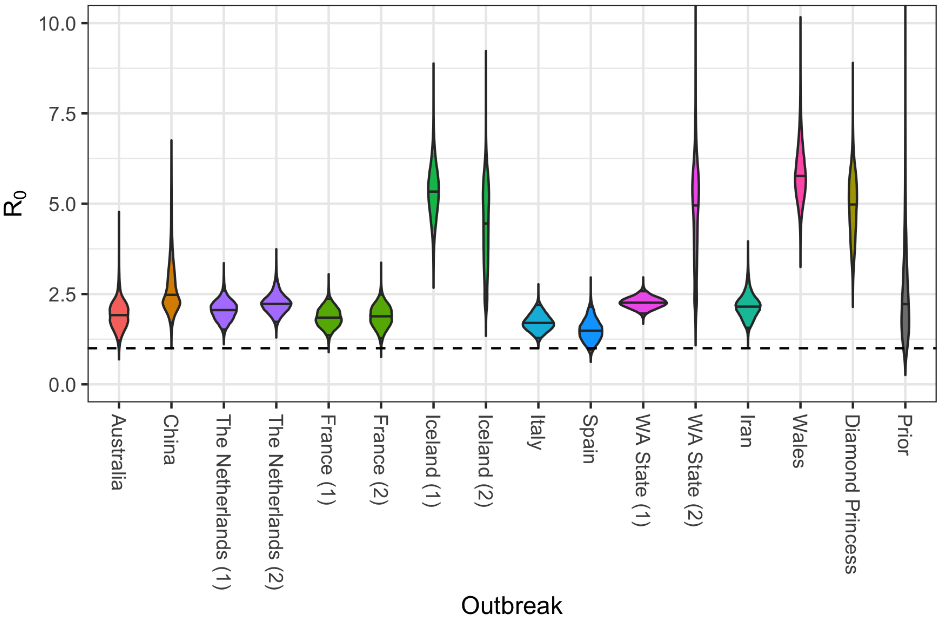

Figure S3: Comparison of R_0 posterior distributions estimated using Bayesian model averaging. (Only the value prior to the quarantine aboard the Diamond Princess was included in this averaging.)

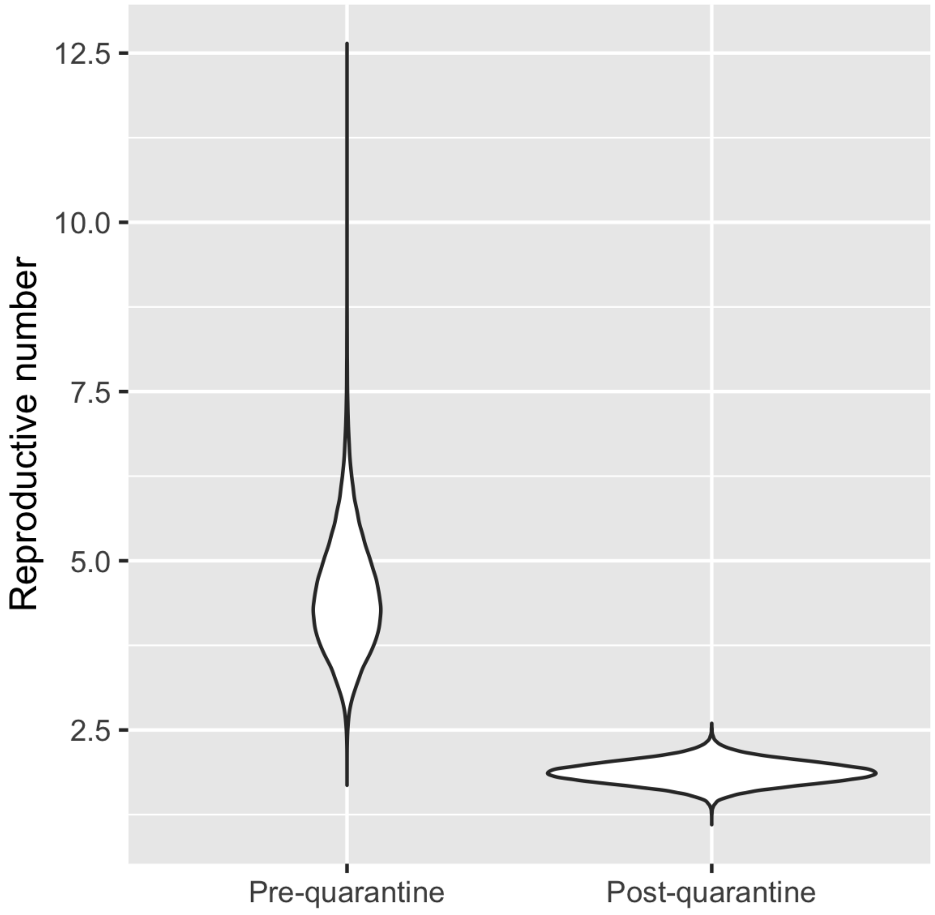

Figure S4: Comparison of R_0 posterior distributions estimated for the pre- and post-quarantine phases of the Diamond Princess outbreak.

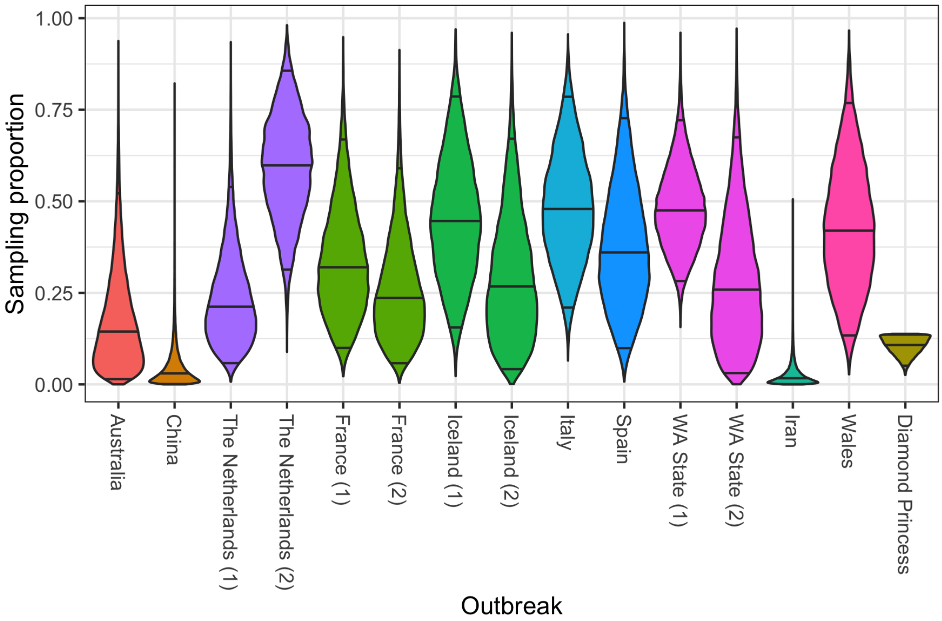

Figure S5: Inferred sampling proportions corresponding to the outbreaks analyzed. Non-informative priors were used for all sampling proportions except for the one corresponding to the Diamond Princess. (See methods).

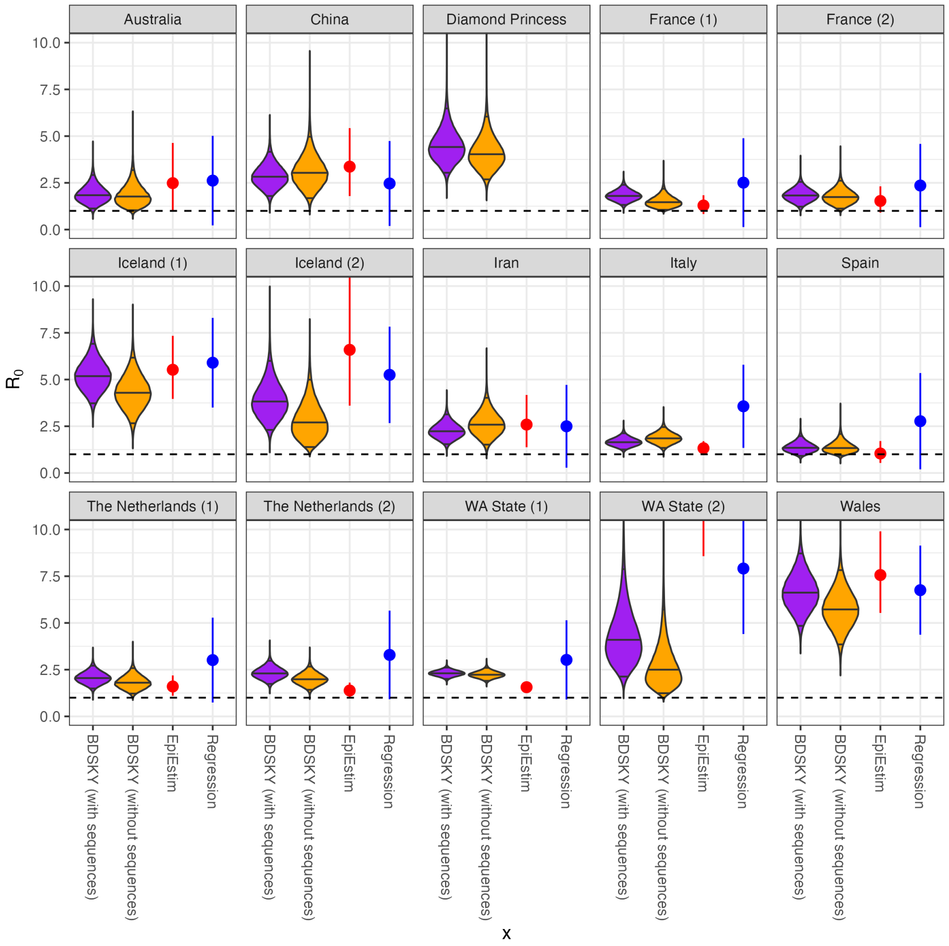

Figure S6: R_0 values inferred using (a) phylodynamic method *with* sequence data, (b) phylodynamic method *without* sequence data, (c) EpiEstim (1) analysis of sequenced sample collection times, and (d) overly simplistic linear regression of outbreak-specific cumulative sample count distributions. (The Diamond Princess regression and EpiEstim results have been excluded from this graphic, as they relate to the post- rather than pre-quarantine phase of that outbreak.)

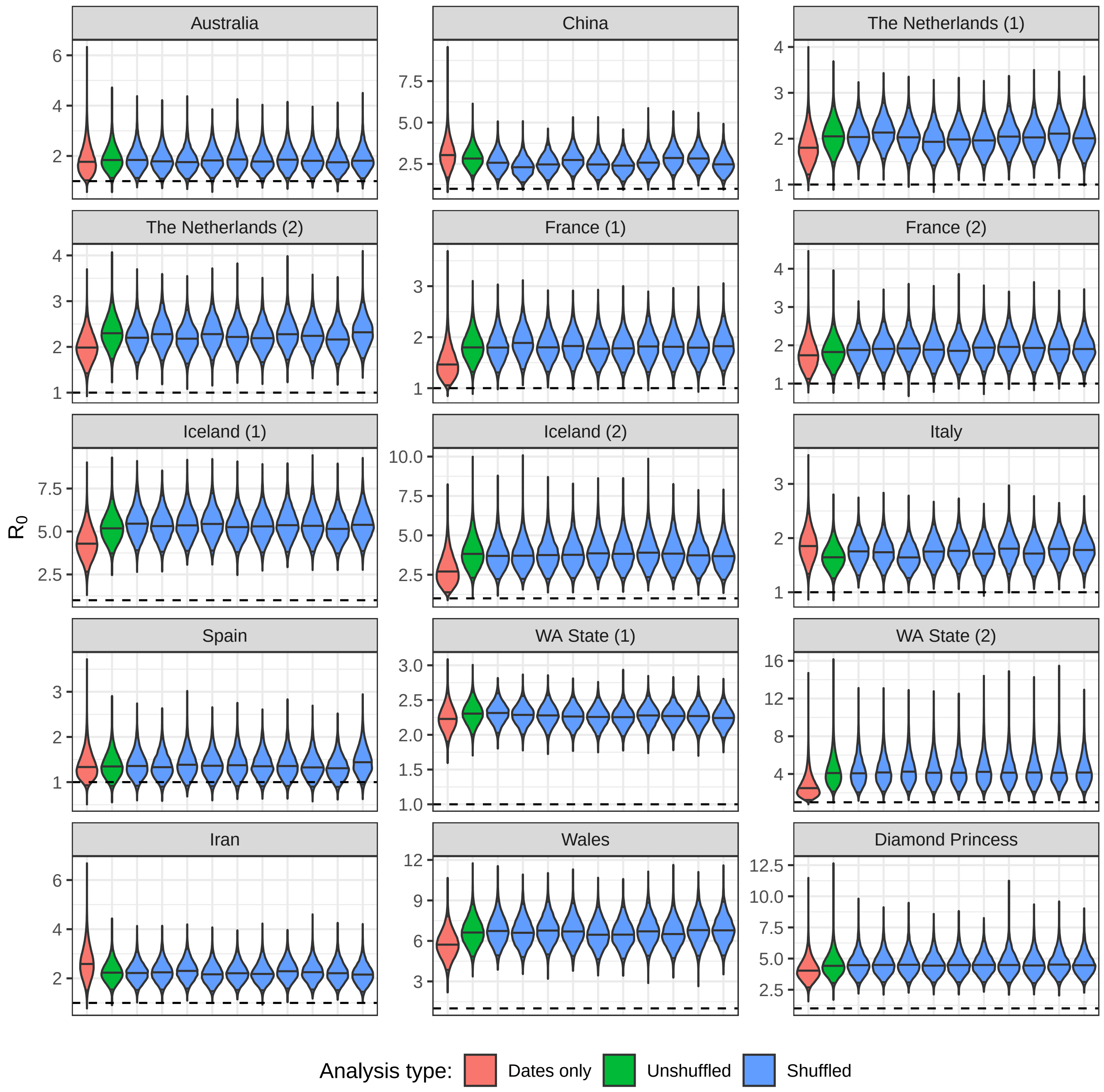

Figure S7: Comparison of phylodynamic R_0 posterior distributions estimated from data sets in which the association between sequences and sample times were randomly shuffled. The posteriors for 10 independently-shuffled data sets are shown (blue), alongside the estimates from the original unshuffled data sets (green) and the estimates from the sequence-free analyses (red).

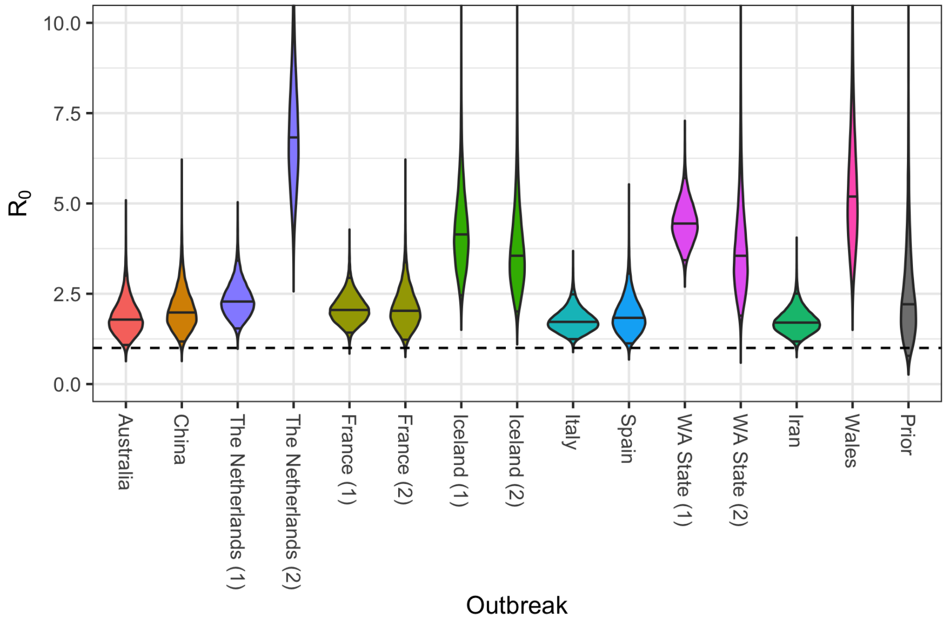

Figure S8: Estimates of R_0 produced using alternative model in which a change in R_0 and the sampling proportion s is permitted at a point midway between the first and last samples of each outbreak. The posteriors shown are for the R_0 values in the earlier of the two intervals.

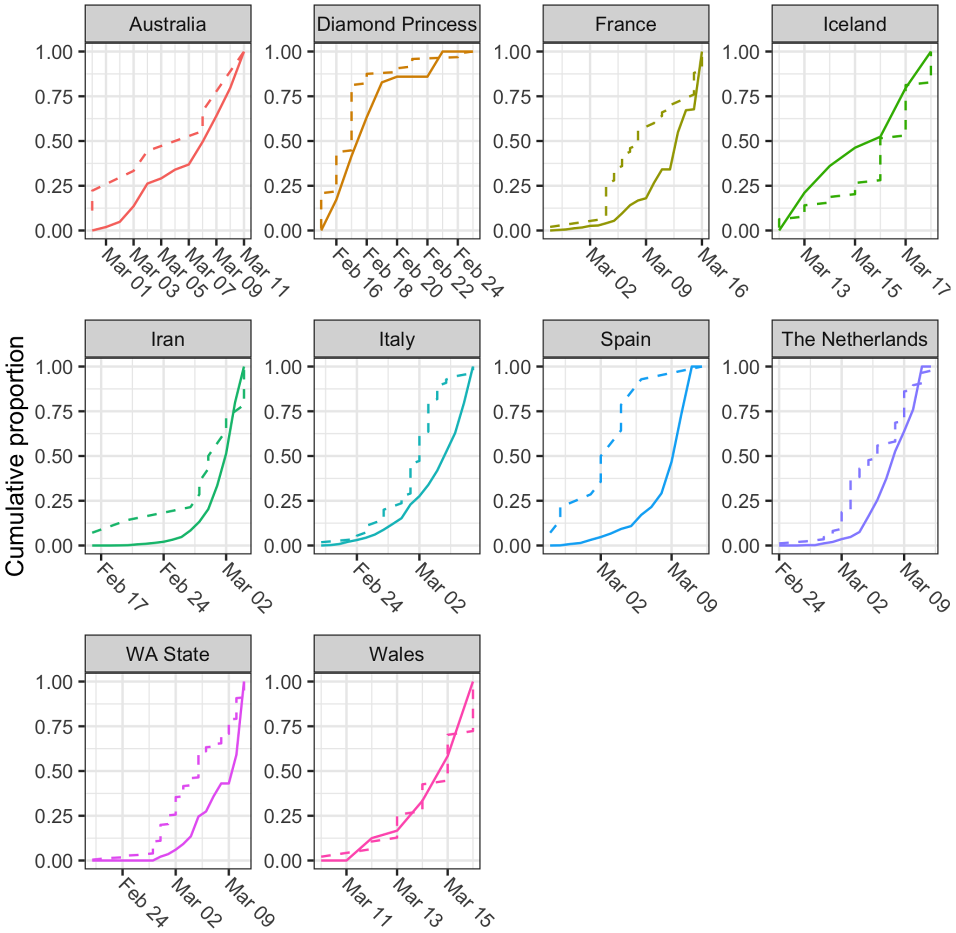

Figure S9: Comparisons between relative cumulative case count (solid) and relative cumulative sequence count (dashed) distributions. These comparisons indicates that our phylodynamic modeling assumption that the included sequences occur at a rate proportional to the pathogen prevalence may be justified in most populations. A possible exception is Spain, which exhibited a higher accumulation of sequences earlier on, compared to the case counts. (Note that the comparison for the Chinese data is not shown, since the collection dates of the Chinese travel sequences do not overlap in time with available case count data specific to the Wuhan province.)

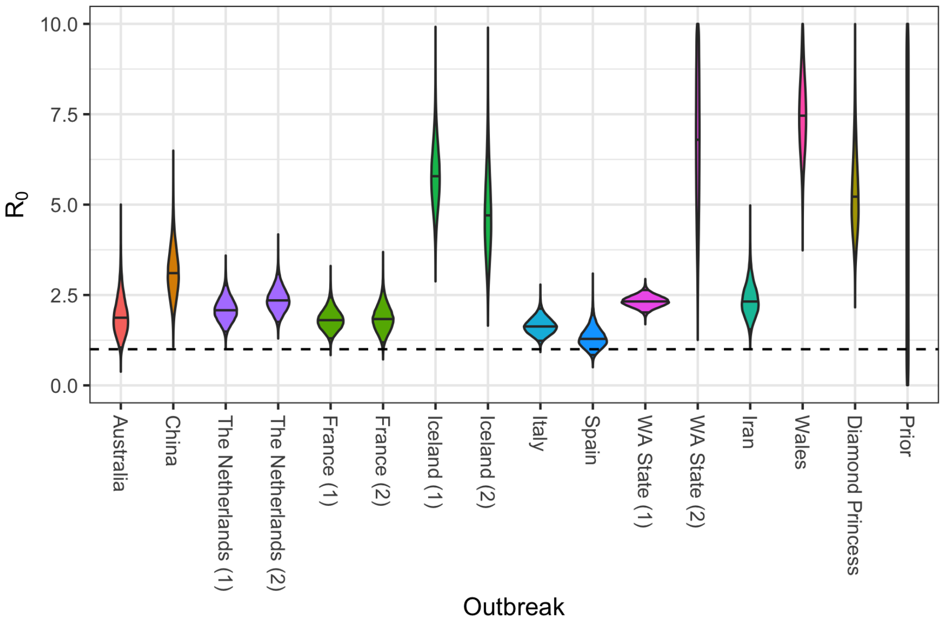

Figure S10: Estimates of R_0 produced using the alternative prior $\text{Unif}(0, 10)$, illustrating the insensitivity of the results to the precise prior used.

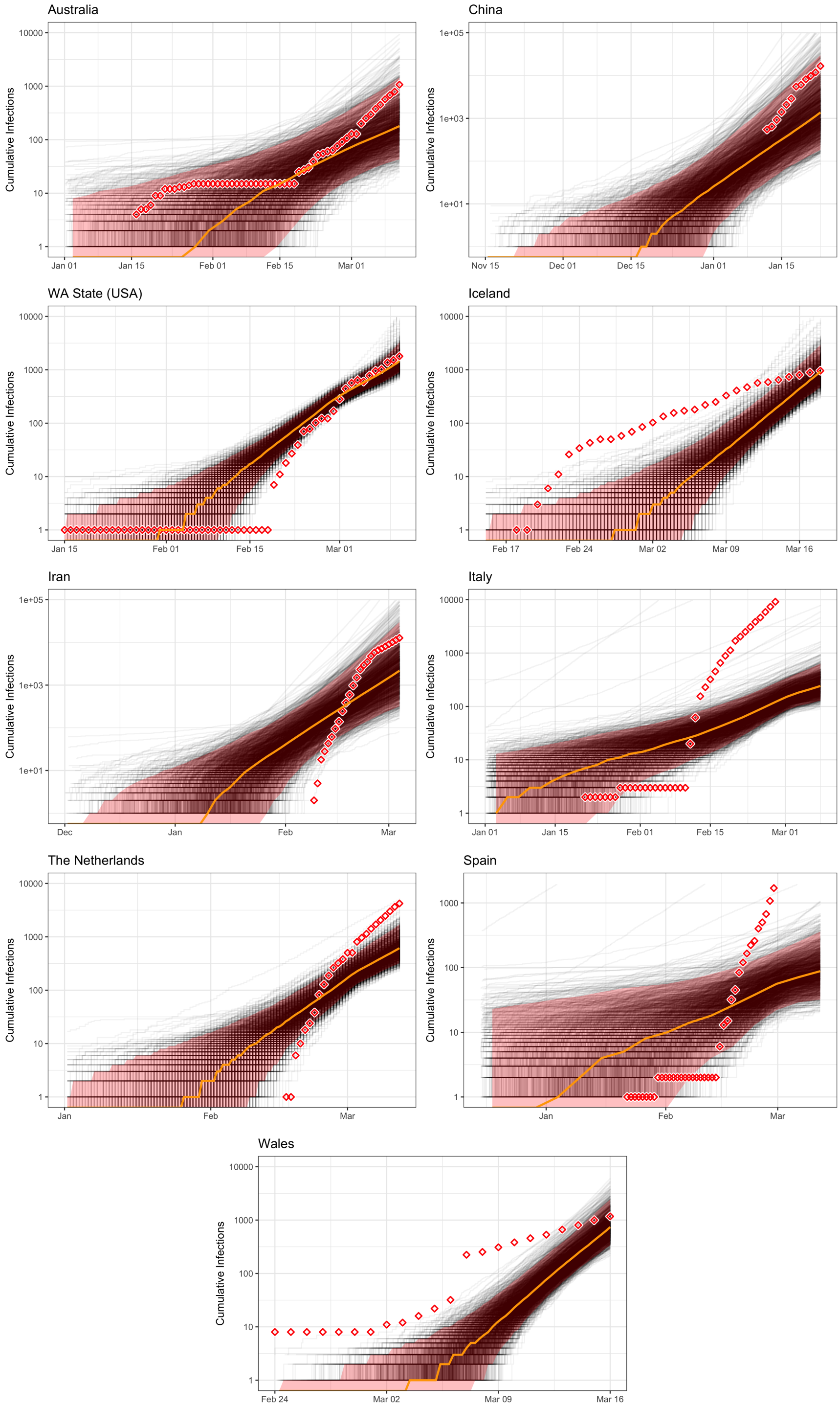

Figure S11: Inferred number of infections for remaining outbreaks.

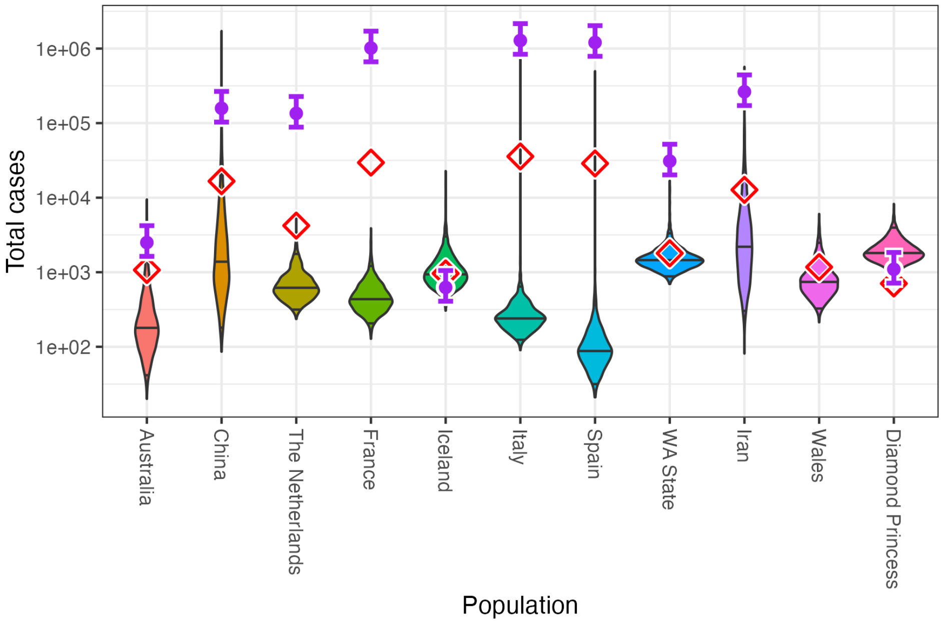

Figure S12: Comparison between estimates of cumulative number of infections obtained from phylodynamic analyses (violin plots), recorded cases (diamonds), and number of infections extrapolated from the cumulative recorded deaths (2) (circles with error bars) using a published estimate (5) of the infection fatality risk (IFR) of 0.64% (95% credible interval $[0.38\%, 0.98\%]$). The IFR-based estimates of the number of infections were time-shifted by -18 days relative to the death statistics to account for both the assumed 10 day delay between infection and positive test results, and a second 8 day delay (4) between positive test results and death. (Welsh IFR-based estimates are not included here, due to the lack of available death statistics in the study period.)

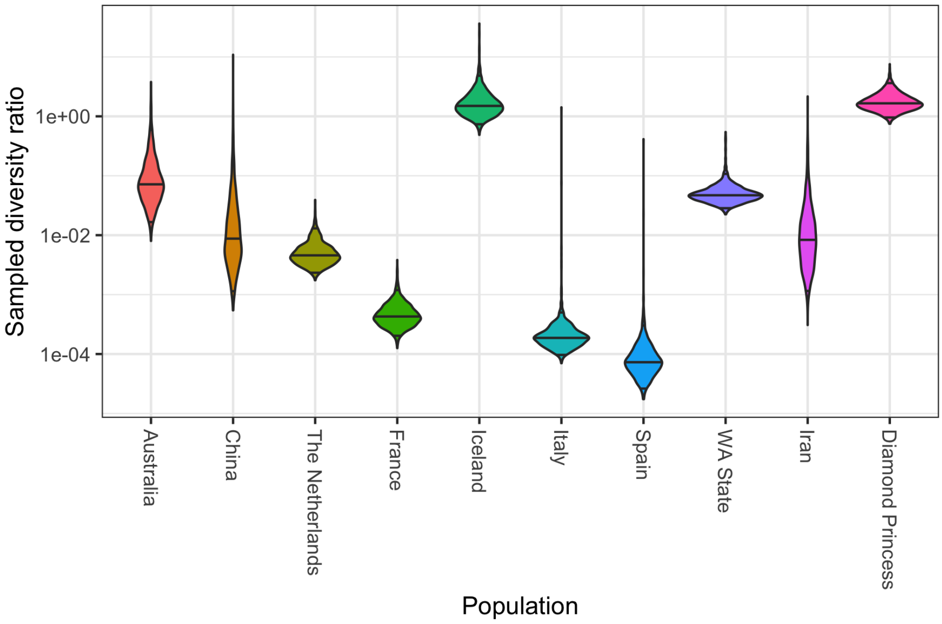

Figure S13: Ratios of phylodynamic estimates of outbreak-specific number of infections to number of infections imputed from recorded death statistics, shown in Figure S12. Assuming the imputed values are accurate, this gives a very approximate indication of the amount of SARS CoV 2 infections in unsequences outbreaks in each population at the study times.

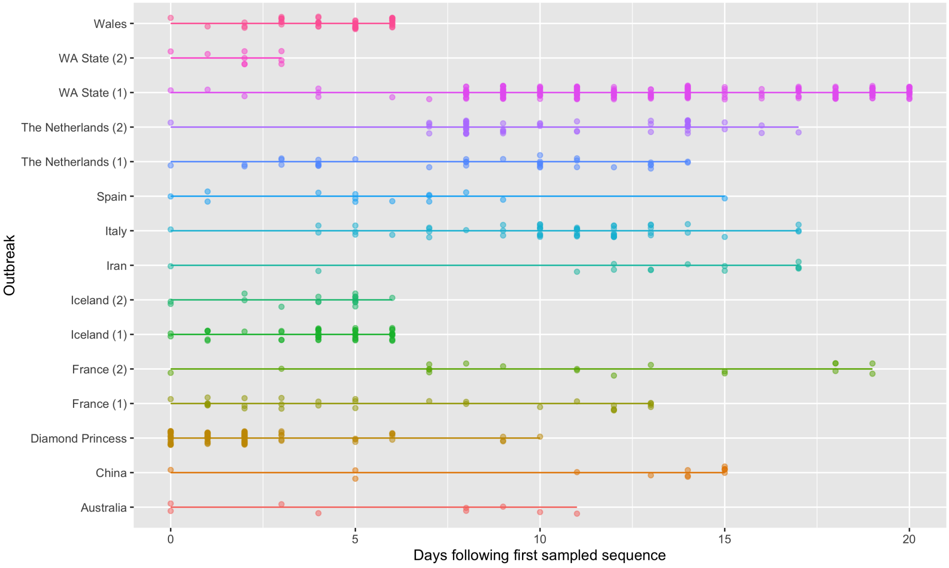

Figure S14: Sample times relative to first sample from each outbreak. Horizontal bars represent full sample period lengths.

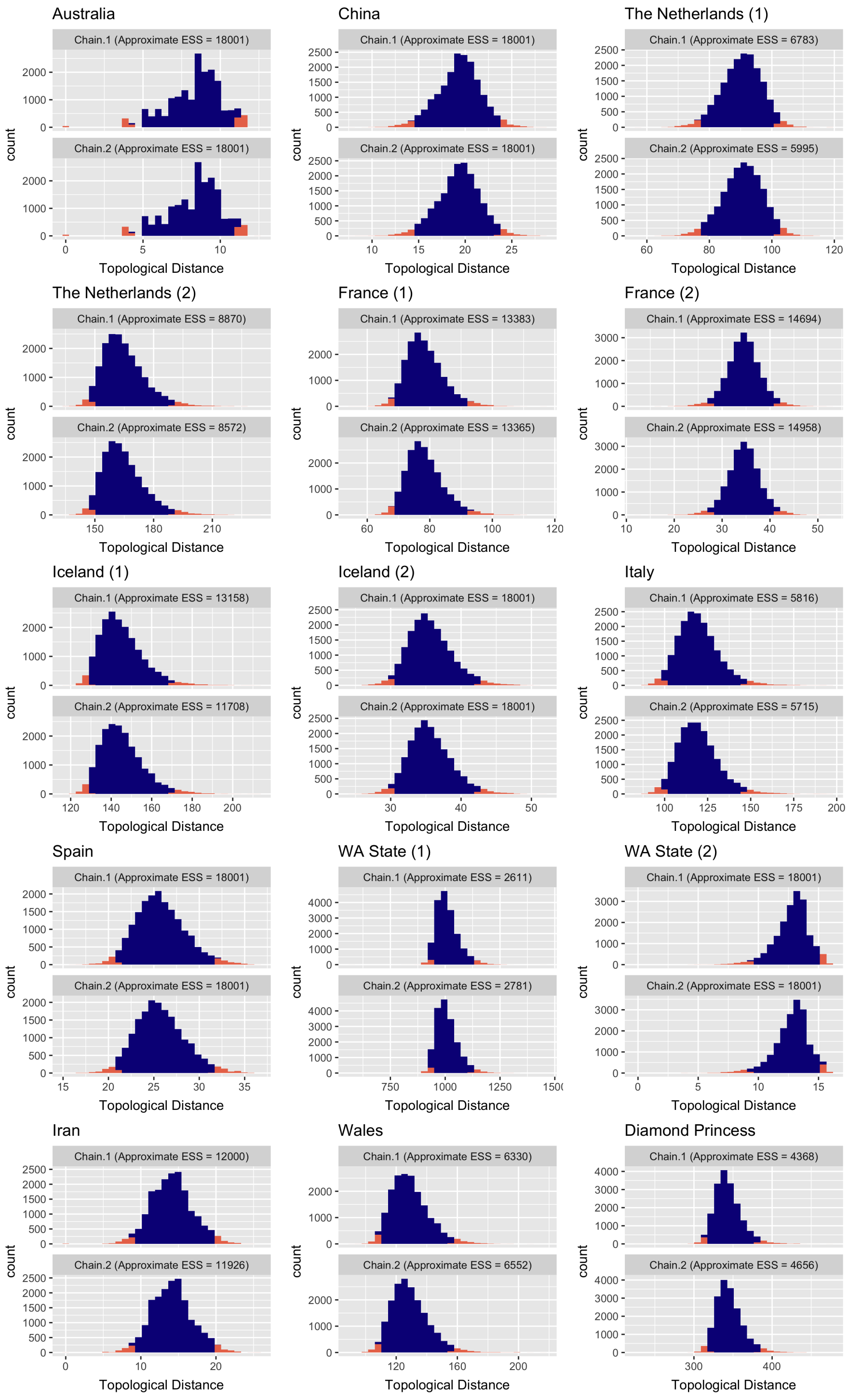

Figure S15: Marginal outbreak-specific tree topology distributions sampled by two independent MCMC chains, where topology is represented as a distance between a given topology and the topology of the first tree (following burn-in) in the first chain. Also shown are the estimated effective sample sizes computed from the distribution.

2 Reversible jump MCMC for Bayesian Model Averaging

To apply the Dirichlet process prior (DPP) defined by equations 1 and 2 in the main text to the cluster-specific $R_0^{(c)}$ values, we use a simple reversible jump Markov chain Monte Carlo algorithm (3).

Specifically, our approach involves the following two modifications to the traditional BDSKY configuration used for analysis described in the section "Main analysis":

1. including a term in the target density corresponding to the prior probability of a particular vector of $\vec{R}_0$ under the DPP, and
2. including an additional state proposal operator allowing the proposal of individual $R_0^{(c)}$ values which are identical to the values corresponding to other clusters.

In our analysis, we used a proposal distribution equivalent to selecting an element of $\vec{R}_0$ uniformly at random, then drawing a new value for that element from its marginal distribution under the DPP:

$$Q(\vec{R}_0'|\vec{R}_0) = \sum_{c=1}^{N} \frac{1}{N} \left[\frac{\alpha}{N-1+\alpha} H(R_0^{(c)}) + \frac{1}{N-1+\alpha} \sum_{i \neq c} \delta(R_0^{(c)} - R_0^{(i)}) \right] \tag{1}$$

where $\vec{R}_0$ is the current set of $R_0^{(c)}$ values, and $\vec{R}_0'$ is the proposed update. (This update scheme would on its own constitute a Gibbs sampler for the prior distribution.)

Despite the change in dimension, the Hastings ratio for this update is given simply by

$$HR(\vec{R}_0'|\vec{R}_0) = \frac{Q(\vec{R}_0|\vec{R}_0')}{Q(\vec{R}_0'|\vec{R}_0)}. \tag{2}$$

References

[1] A. Cori, N. M. Ferguson, C. Fraser, and S. Cauchemez. A new framework and software to estimate time-varying reproduction numbers during epidemics. *American Journal of Epidemiology*, 178(9):1505–1512, sep 2013. doi: 10.1093/aje/kwt133.

[2] E. Dong, H. Du, and L. Gardner. An interactive web-based dashboard to track COVID-19 in real time. *The Lancet Infectious Diseases*, 20(5): 533–534, may 2020. doi: 10.1016/s1473-3099(20)30120-1.

[3] P. Green. Reversible jump Markov chain Monte Carlo computation and Bayesian model determination. *Biometrika*, 82(4):711–732, 1995. doi: 10.1093/biomet/82.4.711.

[4] R. Jin. The lag between daily reported covid-19 cases and deaths and its relationship to age. *Journal of Public Health Research*, 10(3): jphr.2021.2049, jun 2021. doi: 10.4081/jphr.2021.2049.

[5] J. Perez-Saez, S. A. Lauer, L. Kaiser, S. Regard, E. Delaporte, I. Guessous, S. Stringhini, A. S. Azman, D. Alioucha, I. Arm-Vernez, S. Bahta, J. Barbolini, H. Baysson, R. Butzberger, S. Cattani, F. Chappuis, A. Chiovini, P. Collombet, D. Courvoisier, D. D. Ridder, E. D. Weck, P. D'ippolito, A. Daeniker, O. Desvachez, Y. Dibner, C. Dubas, J. Duc, I. Eckerle, C. Eelbode, N. E. Merjani, B. Emery, B. Favre, A. Flahault, N. Francioli, L. Gétaz, A. Gilson, A. Gonul, J. Guérin, L. Hassar, A. Hepner, F. Hovagemyan, S. Hurst, O. Keiser, M. Kir, G. Lamour, P. Lescuyer, F. Lombard, A. Mach, Y. Malim, E. Marchetti, K. Marcus, S. Maret, C. Martinez, K. Massiha, V. Mathey-Doret, L. Mattera, P. Matute, J.-M. Maugey, B. Meyer, T. Membrez, N. Michel, A. Mitrovic, E. M. Mohbat, M. Nehme, N. Noël, H.-K. Oulevey, F. Pardo, F. Pennacchio, D. Petrovic, A. Picazio, G. Piumatti, D. Pittet, J. Portier, G. Poulain, K. Posfay-Barbe, J.-F. Pradeau, C. Pugin, R. B. Rakotomiaramanana, A. Richard, C. R. Fine, I. Sakvarelidze, L. Salzmann-Bellard, M. Schellongova, S. Schrempft, M. S. Miranda, M. Stimec, M. Tacchino, S. Theurillat, M. Tomasini, K.-G. Toruslu, N. Tounsi, D. Trono, N. Vincent, G. Violot, N. Vuilleumier, Z. Waldmann, S. Welker, M. Will, A. Wisniak, S. Yerly, M.-E. Zaballa, and A. Z. Valle. Serology-informed estimates of SARS-CoV-2 infection fatality risk in geneva, switzerland. *The Lancet Infectious Diseases*, 21(4):e69–e70, apr 2021. doi: 10.1016/s1473-3099(20)30584-3.
